# A phase II study of gemcitabine plus nab-paclitaxel as first-line therapy for locally advanced pancreatic cancer

**DOI:** 10.1097/MD.0000000000026052

**Published:** 2021-05-21

**Authors:** Masaru Fukahori, Keisuke Miwa, Kenta Murotani, Yoshiki Naito, Tomoyuki Ushijima, Takahiko Sakaue, Toshimitsu Tanaka, Sachiko Nagasu, Hideya Suga, Tatsuyuki Kakuma, Yoshinobu Okabe, Takuji Torimura

**Affiliations:** a Division of Gastroenterology, Department of Internal Medicine, Kurume University School of Medicine; b Multidisciplinary Treatment Cancer Center, Kurume University Hospital; c Biostatistics Center, Kurume University; d Department of Diagnostic Pathology, Kurume University Hospital, Kurume-shi; e Division of Gastroenterology, Yanagawa Hospital, Chikushi-machi, Yanagawa-shi, Fukuoka, Japan.

**Keywords:** clinical trial, converting to surgical resection, first-line chemotherapy, gemcitabine, nab-paclitaxel

## Abstract

Gemcitabine plus nab-paclitaxel (GnP) is widely used in clinical practice, despite a lack of prospective data to validate its efficacy in locally advanced pancreatic cancer (LAPC). We conducted a phase II study of GnP for LAPC to assess its efficacy and safety.

We performed a single-arm, single-institution study with GnP in 24 patients with LAPC. The treatment protocol included successive administration of gemcitabine (1000 mg/m^2^) and nab-paclitaxel (125 mg/m^2^). The primary endpoint was the tumor overall response rate (ORR), and secondary endpoints were overall survival (OS), progression-free survival (PFS), and adverse events (AEs).

The median PFS was 11.0 months, median OS was 21.2 months, ORR was 62.5%, and 37.5% of the patients had stable disease. Four (16.7%) of the patients were converted to surgical resection; 3 of these achieved R0 resection. Grade 3 to 4 AEs included hematological (neutropenia, 64%; thrombocytopenia, 12%), nonhematological (cholangitis, 16%), and sensory neuropathy (4%). These AEs were manageable and tolerable.

The GnP treatment in patients with LAPC showed favorable tumor shrinkage, good toxicity profile, and enabled conversion to surgical resection in a subset of patients; therefore, GnP is an option for first-line chemotherapy in patients with LAPC.

## 1 Introduction

Pancreatic cancer (PC) is a disease associated with a poor prognosis, with an enhanced impact on cancer-related mortality worldwide.^[1]^ In 2016, approximately 40,000 patients were newly diagnosed with, and 33,000 patients died due to PC in Japan.^[2]^ This disease is not among those with a general trend toward improvement in cancer-related mortality. The 5-year overall survival (OS) rate is reportedly 6.1% for locally advanced pancreatic cancer (LAPC) and only 1.3% for metastatic pancreatic cancer (MPC) in Japan.^[3]^ Therefore, improving the PC prognosis is crucial.

Typically, <10% tumors in patients with PC are resectable and approximately one-third to half are MPC^[4,5]^; the rest are considered LAPC owing to the local invasion of adjacent structures by the cancerous cells. Although surgical resection is the most desirable treatment for PC, the resectability of LAPC depends on the degree of vascular invasion of the superior mesenteric and celiac arteries. If LAPC undergoes shrinkage with an improved degree of invasion of the adjacent major vessels as an effect of systemic treatment, surgical resection of the tumor becomes a possibility.

As a therapeutic strategy, the current National Comprehensive Cancer Network (NCCN) guidelines recommend systemic chemotherapy, chemoradiotherapy (CRT), and induction chemotherapy followed by CRT as the first-line therapy for patients with LAPC. However, it remains unclear which of these treatments is ideal.^[6]^ Recent studies on combination chemotherapy regimens, such as the use of FOLFIRINOX (leucovorin, 5-fluorouracil, irinotecan, and oxaliplatin) and gemcitabine plus nab-paclitaxel (GnP) showed positive outcomes for patients with MPC,^[7–9]^ indicating that both regimens can be considered as the current standard first-line therapy. Particularly in the preclinical study, the combination of nab-paclitaxel and gemcitabine depleted the peritumoral desmoplastic stroma, and intratumoral concentration of gemcitabine increased in mice treated with nab-paclitaxel compared with those receiving gemcitabine alone.^[10]^ The development of combination chemotherapy regimens, at least in cases of metastatic disease, led to significantly higher objective response rates than those observed with gemcitabine monotherapy (39% [FOLFIRINOX]) and 23% [GnP] versus approximately 10% with gemcitabine monotherapy). Therefore, many institutions have embraced induction combination chemotherapy followed by reevaluation for surgical exploration of patients with LAPC.^[7,8]^ However, neither of the regimens has been analyzed prospectively in these patients.

More recently, several clinical evaluations of the therapeutic effects of FOLFIRINOX in patients with LAPC have been reported.^[11–15]^ In contrast, there is a paucity of available reports on the clinical applications of GnP for patients with LAPC, and only 1 broad-spectrum clinical trial report from Western countries is available, excluding Japan.^[16]^ Therefore, we aimed to conduct a phase II clinical trial study in our institution to assess the efficacy and safety of GnP in patients with LAPC.

## 2 Methods

### 2.1 Study design

We conducted a phase II open-label, single-arm, and single-institution study at Kurume University Hospital in Japan from March 2015 through March 2019. The primary endpoint of this trial was to determine the objective or overall response rate (ORR) to predict the clinical benefits experienced by the patients, according to the response evaluation criteria in solid tumors (RECIST) version 1.1 in patients with LAPC treated with GnP. Secondary endpoints were to evaluate OS, progression-free survival (PFS), the disease control rate (DCR), and adverse events (AEs).

### 2.2 Patient selection

Inclusion criteria for the study participants were as follows:

1. diagnosis of pancreatic ductal adenocarcinoma and LAPC (including unresectable only) via histological or cytological confirmation;
2. diagnosis of LAPC by the investigator using imaging criteria established by the NCCN guidelines^[17]^;
3. no prior chemotherapy of any type for the advanced disease;
4. aged ≥20 years;
5. Eastern Cooperative Oncology Group performance status of 0 or 1;
6. no prior radiotherapy; and
7. adequate hematological, renal, and liver function (absolute neutrophil count ≥1500/μL, platelet count ≥100,000/μL, hemoglobin ≥9.0 g/dL, creatinine <1.5 mg/dL, bilirubin <.25 × upper limit of normal [ULN] [if biliary drainage was performed, allowed <3.0 × ULN], and serum aspartate aminotransferase and serum alanine aminotransferase levels of <2.5 × ULN).

Our exclusion criteria were the presence of:

1. distant metastasis;
2. symptomatic pulmonary fibrosis;
3. interstitial pneumonia;
4. active, uncontrolled bacterial, viral, or fungal infections requiring systemic therapy;
5. severe complications (significant cardiac disease, ileus, mental disorder); or
6. pregnancy.

### 2.3 Patients’ treatment

Eligible patients received successive 30-minutes intravenous-infusion cycles of nab-paclitaxel at a dosage of 125 mg/m^2^, followed by a 30-minutes intravenous infusion of gemcitabine 1000 mg/m^2^ on days 1, 8, and 15 every 4 weeks (cycle 1). Treatment was continued except in cases of disease progression, unacceptable AEs, or withdrawal of consent. Additional treatment options, such as surgical resection or CRT, were permitted if good tumor shrinkage was achieved.

### 2.4 Assessments of therapeutic effect

Tumor response was evaluated every 8 weeks (±2 weeks) by computed tomography or magnetic resonance imaging. The pancreatic tumor was assessed by the investigators using RECIST version 1.1.^[18]^ Each patient's complete response (CR) or partial response (PR) required subsequent confirmation of response ≥4 weeks later. ORR was defined as the proportion of patients with CR plus those with PR. DCR was defined as the proportion of patients with CR, PR, and stable disease maintained for ≥4 weeks. PFS was defined as the time from the date of enrollment to the progressive disease or any cause of death; OS was defined as the time from the date of enrollment to any cause of death. Carbohydrate antigen (CA)19-9 level was assessed at baseline and every 4 weeks thereafter. Investigators monitored treatment-related and serious AEs through weekly laboratory testing, the rates of dose reductions, and the relative dose intensity of the drug under study. Treatment-related AEs were graded in accordance with the National Cancer Institute Common Terminology Criteria for Adverse Events version 4.0 (https://evs.nci.nih.gov/ftp1/CTCAE/CTCAE_4.03/CTCAE_4.03_2010-06-14_QuickReference_5x7.pdf).

### 2.5 Statistical analysis

We evaluated the ORR at 80% CIs employing the Clopper–Pearson method. We estimated all summary statistics on time-to-event variables with the Kaplan–Meier method. We estimated the 95% CIs for median survival time and time-specific rate using the Brookmeyer–Crowley method and Greenwood formula, respectively. We estimated that a sample size of minimum 21 patients would be ideal according to a threshold ORR of 15% and an expected ORR of 35% in accordance with the results of previous studies,^[19,20]^ with an alpha value of 0.1 (1-sided) using the binomial test. Given an expected 10% of ineligible patients, we determined the target sample size to be at least 24 patients. We analyzed the ORR and secondary endpoints at the end of the 1-year follow-up period since the last patient enrollment. All enrolled patients were divided into the following 2 chemotherapy groups: patients who received prolonged chemotherapy and patients who received CRT or surgical resection. We statistically compared the median OS between the 2 groups using the log-rank test and performed all statistical analyses with SAS 9.4 (SAS, 9.4, SAS Institute Inc., Cary, NC).

## 3 Results

### 3.1 Patient background

**Figure 1:**
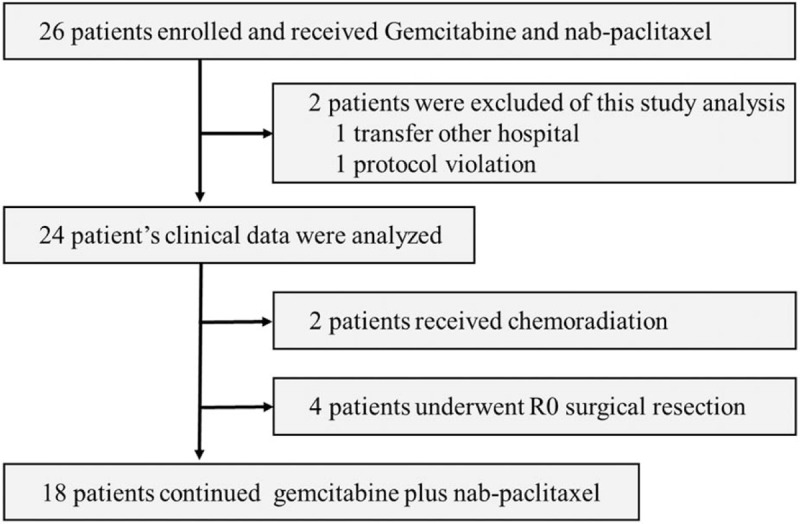
Consort flow chart diagram. Scheme showing enrollment and allocation of locally advanced pancreatic cancer (LAPC) patients in a single-arm, open-label study.

The study profile is displayed as a consort diagram in Figure 1. A total of 26 patients with LAPC met the inclusion criteria in the study; 2 patients were excluded from the study because the ORR of 1 patient was inaccessible due to transfer to another hospital and the other had a protocol violation due to an unconfirmed diagnosis of an unresectable tumor.

Ultimately, 24 patients were considered eligible for the study analysis. Median follow-up time was 12.9 months (range 6.5–55). Demographics and baseline clinical characteristics are listed in Table 1. The median patient age was 68 years (range 44–76). The tumor was located on the pancreatic head/uncinate lesion in 11 (46%) patients and on the body/tail lesions in 13 (54%) patients. The median baseline assessment of the longest diameter of the tumor's target lesion was 32.6 mm (range 23.8–85). The main unresectable factor was an arterial invasion, which was found in 22 (92%) patients. The baseline carbohydrate antigen 19-9 (CA19-9) value was 268 U/ml (range <2.0–1763). The baseline CA19-9 value was elevated from the reference value in 14 (58%) patients.

**Table 1:** Baseline patient characteristics.

Characteristics	N = 24 (%)
Age [yr; median (range)]	68 (44–76)
Sex	
Male	13 (54)
Female	11 (46)
ECOG-performance status	
0	17 (71)
1	7 (29)
Tumor location	
Head/uncinate	11 (46)
Body	11 (46)
Tail	2 (8)
Tumor size [mm; median (range)]	32.6 (23.8–85)
Biliary drainage	
Yes	6 (25)
No	18 (75)
Vascular invasion, main unresectable factor	
CA	11 (46)
SMA	11 (46)
SMV, PV	2 (8)
CA19-9 median (range), U/mL	268 (2.0–1763)

### 3.2 Efficacy

Of 24 patients, the median number of protocol treatment cycles received was 8 (range 3–19). The median percentage of the planned dose the patients received (relative dose intensity: RDI) was 58.9% (range 41.7–99) for gemcitabine 1000 mg/m^2^ and 55.6% (range 41.7–92.7) for nab-paclitaxel 125 mg/m^2^, respectively. None of the patients could tolerate an induction dose or a regulated schedule.

Surgical resection or CRT was performed in 6 patients. Of these, 4 were able to convert to R0 resection, and the median treatment duration until the surgical resection was 9.6 months (range 8.8–10.0). The number of protocol treatment cycles that the patient received was 10 to 11. Two patients received CRT (Fig. 1).

PR was confirmed in 62.5% (95% CI 40.6–81.2, 80% CI 47.4–75.9) based on RECIST (Table 2); therefore, the null hypothesis for the primary endpoint (ORR < 15%) was rejected (*P* < .001). Nine (37.5%) patients achieved stable disease, and none of the patients had CR or disease progression in this study. All patients achieved disease control.

**Table 2:** Objective response rate.

Primary analysis (ORR)		
62.5% (80% CI: 47.4%–75.9%)	*P* < .001	95% CI: 40.6–81.2%
Response during the protocol treatment	N = 24	
Complete response, n (%)	0	95% CI: 0%–14.2%
Partial response, n (%)	15 (62.5)	95% CI: 40.6%–81.2%
Stable disease, n (%)	9 (37.5)	95% CI: 18.8%–59.4%
Progressive disease, n (%)	0	95% CI: 0%–14.2%
DCR, n (%)	24 (100)	95% CI: 85.6%–100%
DCR 4 mo	23 (96)	
DCR 6 mo	18 (75)	

**Figure 2:**
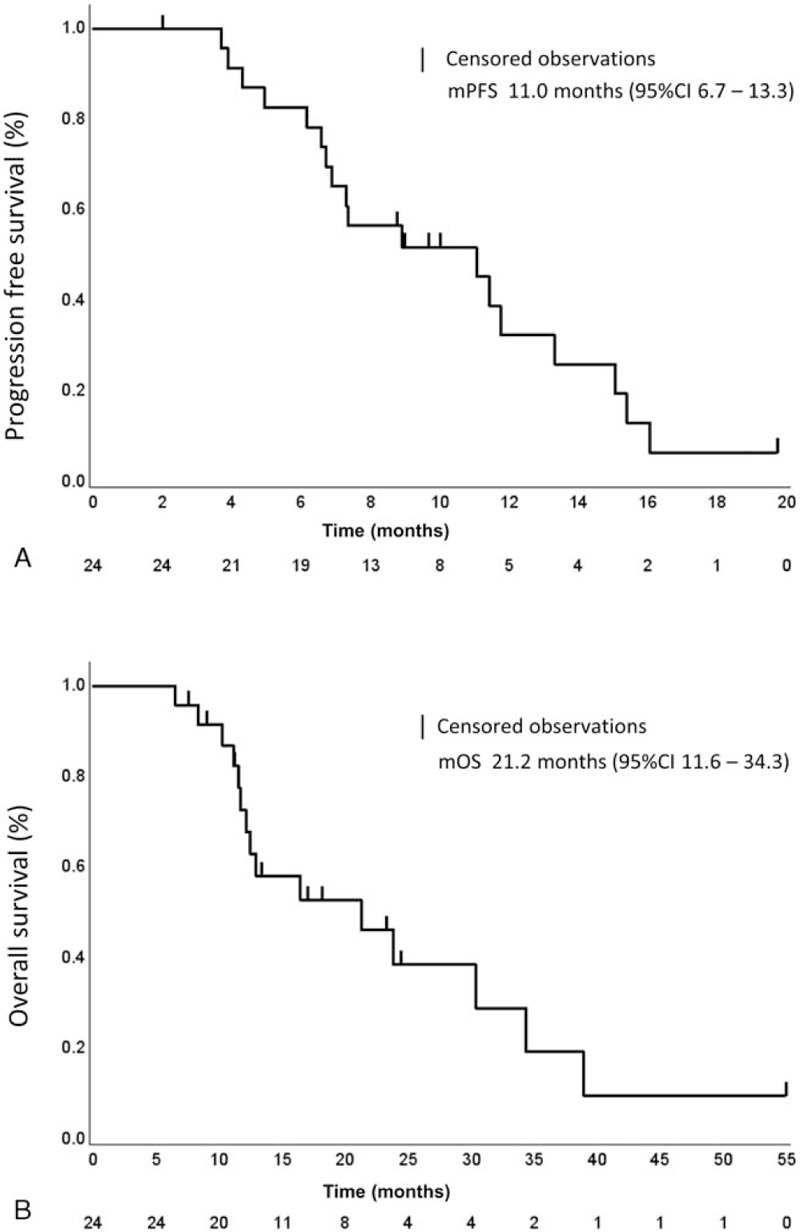
Kaplan–Meier plots of progression free survival (PFS) and overall survival (OS). (A) Kaplan–Meier plots of PFS. (B) Kaplan–Meier plots of OS: data cut-off for survival results was March 31, 2020.

**Figure 3:**
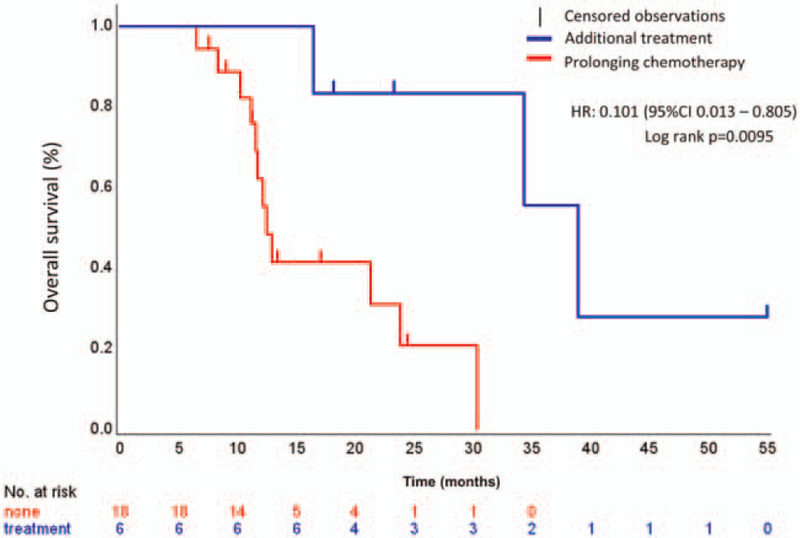
Kaplan–Meier plots of overall survival (OS). Median OS was 12.5 months in prolonging the chemotherapy group and 38.9 months in the additional treatment group.

Median PFS and OS were 11.0 (95% CI 6.7–13.3; Fig. 2A) and 21.2 months (95% CI 11.6–34.3; Fig. 2B), respectively. We compared the survival period between the chemotherapy group and the additional treatment group. Median OS in the additional treatment group (38.9 months; 95% CI 16.4–not reached) was significantly longer than that in the chemotherapy group (12.5 months; 95% CI 11.1–23.8; *P* = .0095; Fig. 3).

**Figure 4:**
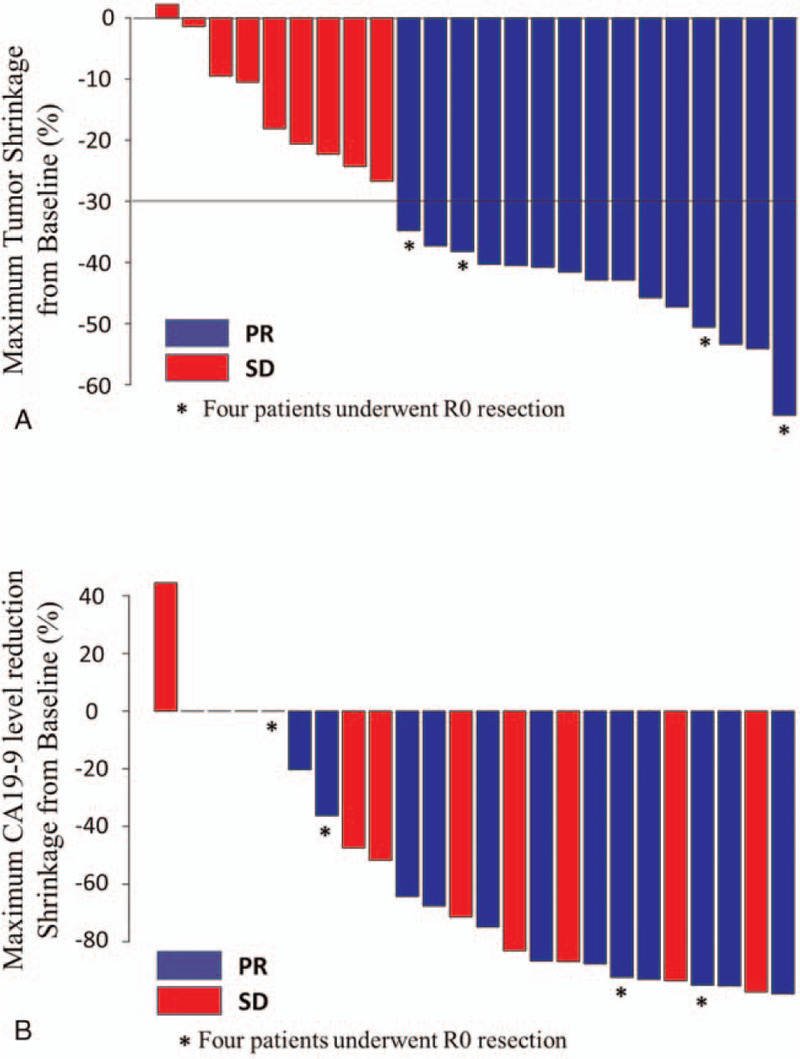
Best percentage change from baseline of the target lesion and maximum percentage in CA19-9 level. (A) Waterfall plot of maximum percent change in tumor size from baseline, as measured according to RECIST. (B) Waterfall plot of maximum percent change in CA19-9 level. Blue bar shows PR and Red bar shows SD in RECIST assessment, respectively. RECIST = response evaluation criteria in solid tumors, SD = stable disease, PR = partial response.

Twenty-three (95.8%) patients with minimum 1 postbaseline assessment showed tumor reduction during the treatment (Fig. 4A). The tendency of an inverse relationship between the CA19-9 value and tumor shrinkage was noticeable, except in 4 patients with CA19-9 levels less than the sensitivity level (Fig. 4B).

### 3.3 Safety

The details of possible AEs that occurred in the patients during treatment are presented in Table 3. Grade 3–4 AEs occurred in 20 (83.3%) patients. The most frequent grade 3 to 4 hematological AEs were neutropenia (64%) and thrombocytopenia (12%). Grade 3 to 4 nonhematological AEs were predominantly infections, including biliary infections in 4 (16%) patients and febrile neutropenia, pyelonephritis, and central venous port infection in 1 (4%) patient. Grade 3 peripheral sensory neuropathy was observed in 1 patient, including 19 (79%) of the grade 2 and below patients. Moreover, there were a few severe cases of typical AEs, including gastrointestinal toxicity, arthralgia, and myalgia.

**Table 3:** Adverse events.

Treatment-related adverse events during the protocol treatment (N = 24), n (%)				
	Grade 1	Grade 2	Grade 3	Grade 4
Hematologic adverse events				
Neutropenia	3 (12)	2 (8)	11 (44)	5 (20)
Anemia	4 (16)	7 (28)	0	0
Thrombocytopenia	4 (16)	8 (32)	3 (12)	0
Non-hematologic adverse events				
Febrile neutropenia	0	0	1 (4)	0
Neutropenic sepsis	0	0	0	1 (4)
Infection				
Cholangitis	0	0	3 (12)	1 (4)
Pyelonephritis	0	0	1 (4)	0
Central vein access port infection	0	0	1 (4)	0
Cerebral infarction	0	0	1 (4)	0
Peripheral sensory neuropathy	10 (40)	8 (32)	1 (4)	0
Fatigue	4 (12)	7 (28)	0	0
Nausea	5 (20)	0	0	0
Appetite loss	7 (28)	1 (4)	1 (4)	0
Constipation	6 (24)	0	0	0
Diarrhea	1 (4)	0	1 (4)	1 (4)
Arthralgia, myalgia	5 (20)	3 (12)	0	0
Alopecia	13 (52)	5 (20)	0	0
Interstitial pneumonia	0	1 (4)	0	0

## 4 Discussion

Complete surgical resection is the only curative option for patients with PC. However, the rate of complete resection is low in patients with LAPC; therefore, systemic chemotherapy is generally recommended to control tumor progression and maintain patients’ quality of life. Until recently, most of the clinical trials for patients with LAPC focused on evaluating the effect of systemic chemotherapy based on single agents (5-fluorouracil, gemcitabine, or S-1) or CRT.^[21–23]^ The results of these conventional therapies were reportedly 5% to 41% for ORR, 4.4 to 8.7 months for median PFS, and 9.2 to 16.8 months for median OS. To the best of our knowledge, no report had described patients with LAPC converting to surgical resection, which implied that treatment outcomes yielded no chance for surgery to remove the cancer in patients with LAPC in that period.

Thereafter, trial LAP07, known to exhibit a typical phase III trials’ reported the therapeutic effect on induction chemotherapy was utilized, followed by CRT for patients with LAPC. Unfortunately, this approach also yielded no significant improvement in survival with gemcitabine or gemcitabine plus erlotinib treatment, followed by CRT.^[24]^ In the present study, only 2 patients received CRT after the GnP chemotherapy; the survival period was 16.4 and 34.3 months, respectively. Both patients had progressive disease with distant metastasis. However, as an interesting outcome, in the LAP07 study, only 18 (4%) of 442 patients converted to surgical resection following induction chemotherapy, despite the absence of criteria for converting to surgical resection. Surprisingly, the median survival periods were significantly improved in these 18 patients (30.9 months), which indicated that the LAPC-converted surgical resection patients achieved a longer survival period than those who could not convert.^[24]^

Recently, several clinical trials of FOLFIRINOX for LAPC have been published. A meta-analysis of FOLFIRINOX, including 13 studies of 315 patients with LAPC, demonstrated that FOLFIRINOX contributed to the improvement of the survival period by enabling conversion to surgical resection at a higher rate than that of LAP07 (resection rate 0%–43%, R0 resection rate 74%, median OS 10–32.7 months, with a patient-level median OS of 24.2 months).^[25]^

Most recently, Philip et al^[16]^ reported, possibly for the first time, a prospective phase II study (LAPACT) of GnP for patients with LAPC. They reported an ORR of 34.0%, DCR of 90.6%, median PFS of 10.9 months (90% CI 9.3–11.6), and a median OS of 18.8 months (90% CI 15.0–24.0) for 106 patients with LAPC using GnP as induction chemotherapy. The therapeutic effect might be equal to or better than that of conventional CRT. Findings of the present study indicated that ORR was 62.5%, DCR was 100%, median PFS was 11.1 months, and median OS was 21.3 months, thereby reproducing the results of the LAPACT study.

Moreover, the LAPACT study reported that 15.1% (16/106) of the patients were able to convert to surgical resection.^[16]^ This trial indicated that GnP allows a certain number of patients with LAPC to convert to surgical resection following induction chemotherapy in addition to FOLFIRINOX. The present study also enabled 16.7% (4/24) of the patients to convert from unresectable to surgically resectable disease, which showed that the drug efficacy was equivalent to that of the LAPACT study. Three of the 4 patients converting to surgical resection achieved R0 resection with no recurrence thus far.

As mentioned above, recent data, which included the present study results, have indicated that qualification for converting to surgical resection has great potential for further improving the survival period in patients with LAPC. The American Society of Clinical Oncology and NCCN guidelines indicate the importance of converting to surgical resection as a success of induction chemotherapy; however, there was no concrete mention of the criteria for conversion.^[26]^

Furthermore, there is concern regarding difficulties in determining the indication to convert to surgical resection in patients with LAPC. A retrospective study of the patients with LAPC or borderline PC treated with gemcitabine, based on the combination chemotherapy, indicated a reduction of minimum 50% of the PC management marker, CA19-9, which might help identify patients likely to benefit from surgical resection after induction chemotherapy.^[27]^ In the present study, 3 of 4 patients who converted to surgical resection showed CA19-9 elevation at baseline. Among these, although 2 patients exhibited CA19-9 reduction tendencies, 1 showed no apparent reduction in CA19-9 levels, which was <50% after the first-line chemotherapy. However, the exact cut-off value of serum CA19-9 to consider when converting to surgical resection remains unclear. Nonetheless, it is imperative to make the best possible decision when converting to surgical resection in patients with LAPC to determine surgical indication. Thus, it has been recommended to conduct a laparoscopic examination to support the decision, as recently reported.^[28]^

Regarding AEs, the incidence of grade 3 to 4 AEs occurred in 20 (83%) of 24 patients during the protocol period of this study, whereas only 1 (4%) patient discontinued chemotherapy due to interstitial pneumonia, which was generally consistent with the phase III MPACT and Japanese phase II trials^[8,29]^ and within our expectations. We compared the AEs of our study with those of FOLFIRINOX. Both regimens showed hematological toxicities, especially neutropenia. Regarding non-hematological toxicity, such as gastrointestinal toxicity and fatigue, they were less frequent than with FOLFIRINOX^[7]^. However, it should be noted that neutropenia and peripheral sensory neuropathy are common AEs in GnP.

Furthermore, the LAPACT study revealed that 20.7% of the patients discontinued chemotherapy during the induction phase due to AEs. The RDIs were 84.2% for nab-paclitaxel and 82.2% for gemcitabine. Conversely, although our results revealed an RDI of <60%, only 1 patient discontinued chemotherapy; the rest obtained good tumor shrinkage and disease control. Considering the present data were equivalent to that of the LAPACT study, appropriate dose reduction and drug withdrawal might permit continuing with the GnP regimen; however, further study is required to clarify this point.

Although the current study yielded favorable outcomes regarding the efficacy and safety of the GnP regimen, there are some limitations. First, given that we performed a single institutional phase II trial, the small sample size might produce enrollment bias in patient selection. Second, we did not set a strict period of time for the induction phase in the present protocol. Provided that the duration of the induction period is set in a restricted fashion, we might be able to clarify the optimal timing to transit the additional treatments and reduce treatment selection bias.

In conclusion, among locally advanced pancreatic cancers, this study is one of the few reports focusing on only unresectable cases. First-line chemotherapy with GnP for our patients with LAPC demonstrated favorable tumor shrinkage, disease control, and a good safety profile. The therapeutic effect of GnP for LAPC is comparable with that of FOLFIRINOX. Regarding AEs, GnP has a lower frequency of non-hematological toxicity, such as gastrointestinal toxicity, than FOLFIRINOX. Thus, it might not reduce the patient's quality of life. Our present prospective clinical trial therefore indicates that GnP could be an option for patients with LAPC. A meta-analysis of GnP therapy for LAPC, including the results of LAPACT and this study, will provide further findings.

## Acknowledgments

The authors are grateful to the patients who participated in this study, their families, and all the participating investigators, including Yusuke Ishida, Kei Kuraoka, Yu Sasaki, Makiko Yasumoto, Yutaka Shimamatsu, Yasutaka Shimotsuura, Hiroto Ishikawa, Tetsuya Nakashima, Hitoshi Obara, Etsuyo Ogo, for assistance with this study. Medical writing assistance was provided by Suguru Fukahori. The authors would like to thank Enago for the English language review.

## Author contributions

**Conceptualization:** Masaru Fukahori, Keisuke Miwa, Hideya Suga, Tatsuyuki Kakuma, Yoshinobu Okabe, Takuji Torimura.

**Data curation:** Masaru Fukahori, Keisuke Miwa, Takahiko Sakaue, Toshimitsu Tanaka, Sachiko Nagasu.

**Formal analysis:** Masaru Fukahori, Kenta Murotani, Tatsuyuki Kakuma.

**Investigation:** Masaru Fukahori, Keisuke Miwa, Tomoyuki Ushijima, Hideya Suga.

**Methodology:** Masaru Fukahori, Keisuke Miwa, Yoshiki Naito.

**Project administration:** Masaru Fukahori, Keisuke Miwa.

**Resources:** Masaru Fukahori.

**Software:** Masaru Fukahori.

**Supervision:** Masaru Fukahori, Keisuke Miwa, Takuji Torimura.

**Validation:** Masaru Fukahori.

**Visualization:** Masaru Fukahori.

**Writing – original draft:** Masaru Fukahori.

**Writing – review & editing:** Masaru Fukahori, Keisuke Miwa, Takuji Torimura.
